# Prognostic Significance of Neutrophil-to-Lymphocyte Ratio in Patients with Sepsis: A Prospective Observational Study

**DOI:** 10.1155/2016/8191254

**Published:** 2016-03-24

**Authors:** Xuan Liu, Yong Shen, Hairong Wang, Qinmin Ge, Aihua Fei, Shuming Pan

**Affiliations:** Department of Emergency, Xinhua Hospital, Shanghai Jiaotong University School of Medicine, 1665 Kongjiang Road, Shanghai 200092, China

## Abstract

*Background*. The neutrophil-to-lymphocyte ratio (NLR) is an easily accessible biological marker that has been reported to represent disease severity. The aim of this study is to investigate the association between NLR and mortality in patients with sepsis.* Methods*. A total of 333 consecutive adult patients with sepsis were screened for eligibility in this prospective, observational study cohort. Severity scores and leukocyte counts were prospectively recorded upon entry to the intensive care unit (ICU). Receiver operating characteristic (ROC) curves and binary logistic regression models were used to assess the performance of NLR in predicting unfavorable outcome. Correlations between variables and disease severity were analyzed through Spearman correlation tests.* Results*. Median NLR levels were significantly higher in patients who died than in survivors. NLR had a modest power for predicting poor outcome as suggested by area under the curve (AUC) of 0.695 ± 0.036. Multivariate linear regression indicated that increased NLR levels were related to unfavorable outcome independently of the effect of possible confounders. Spearman correlation tests showed that there was a positive correlation between NLR levels and disease severity.* Conclusions*. Increased NLR levels were independently associated with unfavorable clinical prognosis in patients with sepsis. Further investigation is required to increase understanding of the pathophysiology of this relationship.

## 1. Introduction

Sepsis is a complicated condition and still a big challenge to both the developed and developing world. The reported morbidity of sepsis is constantly increasing, with severe sepsis and septic shock remaining among the major causes of death worldwide [1]. Although the mortality has been on the decline in recent years [2], low awareness, late identification, and improper management are still common [3]. Studies have found that one of the fundamental principles for the appropriate management of sepsis is early and accurate detection of the patients at high risk for death [4]. This is generally dependent on the application of scoring systems. Although various clinical biomarkers are widely explored [5–8], only a few have been currently applied in the clinical practice. Therefore, the search continues for preferable infection markers that may facilitate the prognosis prediction of sepsis.

The neutrophil-to-lymphocyte ratio (NLR), as a readily accessible biomarker, can be calculated based on a complete blood count. Although a growing body of evidence has shown that NLR is proposed as an independent predictor of poor survival in various clinical circumstances ranging from oncological patients [9, 10] to patients with cardiovascular diseases [11], there is no consensus about the relationship between NLR levels and clinical prognosis in patients with sepsis until now. In the context of infection, researchers in a recent study showed a reversed NLR evolution according to the timing of death [12], whereas some other studies suggested that NLR was not associated with mortality in patients with sepsis [13]. Consequently, the clinical usefulness of NLR in patients with sepsis is therefore still a matter of ongoing controversy and this question deserves further investigation.

In this prospective observational study, we sought to evaluate the potential association of NLR on intensive care unit (ICU) admission with the clinical prognosis in a consecutive series of adult patients with sepsis.

## 2. Material and Methods

### 2.1. Study Design

This prospective trial recruited consecutive adult patients with sepsis admitted to the ICU of the Department of Emergency, Xinhua Hospital, Shanghai Jiaotong University School of Medicine, from October 2013 to October 2015.

For each patient with suspected infection, a complete diagnostic work-up was performed. The work-up comprised demographic and clinical characteristics, conventional risk factors, and important laboratory data including leukocyte counts, blood biochemistry, blood cultures, urine cultures, chest X-ray, and chest or abdominal computed tomography if necessary. Broad spectrum antimicrobial therapy was administered within 1 hour from the recognition of the septic status, always after collecting samples for microbiological culturing.

The inclusion criteria were as follows in the study: (1) age of at least 18 years; (2) sepsis due to one of the following infections: community acquired pneumonia, hospital acquired pneumonia, ventilator-associated pneumonia, acute pyelonephritis, intra-abdominal infection, or primary bacteremia; and (3) blood sampling within 24 hours from the presentation of signs of sepsis. We formulated a priori criterion to exclude patients according to the following criteria: (1) missing neutrophil and lymphocyte data on ICU admission; (2) missing covariate data for multivariable adjustments; (3) patients with immunosuppressive diseases mainly including cancer and HIV infection or patients with receiving immunosuppressive therapy; and (4) patients who were already in ICU for many days and became septic secondary. Patients were eligible for the final study cohort if they met the inclusion criteria and none of the exclusion criteria.

All the eligible patients were further classified according to standard definitions of sepsis, severe sepsis, and septic shock [14]. Specifically, sepsis was defined as the presence of infection together with systemic manifestations of infection; severe sepsis was defined as sepsis with sepsis-induced organ dysfunction or tissue hypoperfusion; septic shock was defined as sepsis-induced hypotension persisting despite adequate fluid resuscitation.

The study was approved by Shanghai Jiaotong University Xinhua Hospital Ethics Committee and was carried out in accordance with the Declaration of Helsinki. All patients were informed about the study and consented to participate. If the patient was unable to be informed, the next of kin was informed and provided consent for the patient to participate.

### 2.2. Blood Measurements

Venous blood (3 mL) was collected from patients presenting to the ICU. The blood was drawn into an EDTA-containing tube (BD Vacutainer, Plymouth, UK) and centrifuged at 3,000 rpm for 15 min, and plasma was frozen at −80°C until analysis. Complete blood count was determined using the Beckman Coulter LH-750 Hematology Analyzer (Beckman Coulter, Inc., Fullerton, California). NLR was calculated as a ratio of circulating neutrophil and lymphocyte counts. The normal ranges for the leukocyte in our laboratory are 1.4–6.5 × 10^9^/L for neutrophil count and 1.2–3.4 × 10^9^/L for lymphocyte count.

### 2.3. Disease Severity and Outcome

To evaluate the severity of sepsis upon presentation, the validated Acute Physiology and Chronic Health Evaluation II (APACHE II) score was calculated in all enrolled patients on admission. This score ranges from 0 to 71, with higher scores indicating more severe disease.

Furthermore, patients who survived and discharged from hospital were further followed up by telephone calls. The primary outcome of the study was defined as death from any cause within 28 days after admission to the ICU.

### 2.4. Statistical Analysis

Continuous variables were reported as mean values ± standard deviation (SD) or median with interquartile range (IQR), while categorical variables were expressed as count and percentage. The statistical significance of intergroup differences was compared through unpaired Student's *t*-test or Mann-Whitney *U* test for continuous variables and through Pearson's *χ* ^2^ test for categorical variables. The ability of the variables to discriminate survivors from nonsurvivors was determined using receiver operating characteristic (ROC) curves. ROC curves showed sensitivity versus 1 − specificity such that area under the curve (AUC) varied from 0.5 to 1.0, with increased values demonstrating higher discriminatory ability. Univariate logistic regression analyses were performed to separately examine the association between unfavorable outcome and each of the indicators. We also conducted forward stepwise multivariate logistic regression models to determine the independent predictors adjusted for the previously specified baseline covariates. Criteria of *P* < 0.05 for entry and *P* ≥ 0.10 for removal were imposed in this procedure. Correlations between variables and APACHE II score were analyzed through Spearman correlation tests. Two-sided *P* value < 0.05 was considered to represent a statistically significant difference. All analyses were performed by the IBM SPSS Statistics software version 19.0 (SPSS, Chicago, Illinois, USA).

## 3. Results

### 3.1. Baseline Characteristics of the Study Population

During the study period, there were 333 consecutive patients (56.46% male; mean age, 70.26 ± 15.79 years) with complete neutrophil and lymphocyte data available, and all of these patients had complete data available for the primary outcome. A total of 253 patients survived and 80 died within 28 days after admission. The baseline clinical and laboratory characteristics of the patients are elaborated in Table 1. The median APACHE II score was 11 (IQR, 6 to 19). 137 patients (41.14%) had sepsis, 149 patients (44.74%) had severe sepsis, and the remaining 47 patients (14.11%) had septic shock. 42 patients (12.61%) received mechanical ventilation treatment, and 24 patients (7.21%) received renal-replacement therapy. The commonest locations of infection were lung and abdomen, and the distribution of locations was similar among survivors and nonsurvivors. There was not any difference in NLR levels between groups with sepsis of pulmonary versus abdominal origin. The commonest isolated pathogens from the study cohort were Gram-negative microorganisms with a predominance of* Escherichia coli*, and blood cultures were positive in 33.63% of all patients. There were 67 bacteremic patients among the survivors and 45 bacteremic patients among the nonsurvivors. NLR levels of the patients with positive blood culture were significantly higher than the ones with negative blood culture (22.65 (IQR, 12.60 to 36.93) versus 14.66 (8.15 to 25.62), *P* = 0.000). Although the median length of stay in the hospital was similar between survivors and nonsurvivors (*P* = 0.468), the median length of stay in the ICU was significantly longer in nonsurvivors (*P* = 0.041). In addition, the proportion of nonsurvivors receiving mechanical ventilation or renal-replacement therapy was greater than survivors (*P* = 0.000).

The median NLR for the entire cohort was 17.85 (IQR, 9.61 to 28.19). The neutrophil count of nonsurvivors on admission was higher than that of survivors (*P* = 0.001). Of note, the lymphocyte count was much less (*P* = 0.002) with an increased NLR (*P* = 0.000) in the nonsurvivors compared to patients that survived. Nonsurvivors tended to be older and have higher baseline levels of APACHE II score, as well as more white blood cell (WBC) count compared with the survivors. However, there was no statistically significant difference in nonsurvivors versus survivors with respect to other conventional infection markers including procalcitonin (PCT) (*P* = 0.521) and C-reactive protein (CRP) (*P* = 0.140).

### 3.2. Value of Indicators in Predicting Unfavorable Outcome

**Figure 1:**
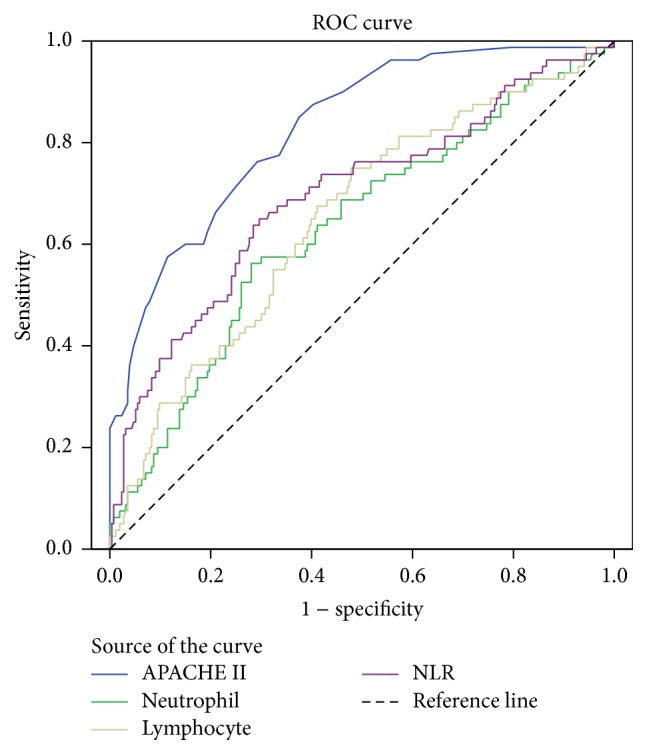
Receiver operating characteristic (ROC) curves for Acute Physiology and Chronic Health Evaluation II (APACHE II) score, neutrophil, lymphocyte, and neutrophil-to-lymphocyte ratio (NLR). NLR had a modest power for predicting unfavorable outcome as suggested by area under the curve (AUC) of 0.695 ± 0.036, *P* = 0.000.

ROC curves were constructed to evaluate the performance of indicators in differentiating nonsurvivors from survivors, and the AUC for each indicator was compared. The AUC, optimal cutoff value, sensitivity, and specificity of each indicator are presented in Table 2. NLR had a modest power for predicting unfavorable outcome as suggested by AUC of 0.695 ± 0.036, which was less than that of baseline APACHE II score (0.828 ± 0.026) but greater than that of neutrophil (0.633 ± 0.036) and lymphocyte (0.650 ± 0.035). NLR ≥ 23.8 was proposed as the optimal cutoff value, which provided a sensitivity of 81.3% and a specificity of 53.6% for predicting mortality in sepsis (Figure 1).

Furthermore, we performed univariate logistic regression analyses to examine the associations of each variable with unfavorable outcome and calculated the standardized regression coefficient (*β*) and the odds ratio (OR) for each variable. As shown in Table 3, baseline APACHE II score had the greatest absolute value of standardized *β* value (0.2342). The absolute value of standardized *β* value for NLR was 0.0378 and the unadjusted OR was 1.038 (95% confidence interval (CI), 1.008–1.070, *P* = 0.013), indicating that NLR had a power for predicting unfavorable outcome.

### 3.3. Independent Prognosis Significance of NLR

We conducted a forward stepwise multivariate logistic regression model to determine the independent predictors of adverse outcome. The results are shown in Table 4. Baseline APACHE II score (adjusted OR, 1.168; 95% CI, 1.108–1.230; and *P* = 0.000) and NLR (adjusted OR, 1.043; 95% CI, 1.012–1.083; and *P* = 0.016) were the independent predictors which entered the final prediction model, indicating that higher NLR levels increased the risk of shifting to an unfavorable outcome independently of the effect of possible confounders. In addition, old age was also independently related to unfavorable outcome (adjusted OR, 1.077; 95% CI, 1.034–1.122; and *P* = 0.000).

### 3.4. Association between NLR and Disease Severity

Patients with severe sepsis or septic shock tended to have higher baseline levels of APACHE II score, neutrophil count, and NLR, as well as lower lymphocyte count compared with patients with sepsis (Table 5). As APACHE II score increased, neutrophil count and NLR levels consistently increased, while lymphocyte count consistently decreased. Furthermore, the correlations between neutrophil count, lymphocyte count, NLR, and APACHE II score were analyzed through Spearman correlation tests. A positive correlation was reported between NLR levels and APACHE II score at baseline (*r* = 0.641, *P* = 0.000), suggesting that NLR levels were positively proportional to disease severity. In addition, neutrophil count was positively correlated with disease severity (*r* = 0.383, *P* = 0.000), while lymphocyte count was inversely proportional to disease severity (*r* = −0.474, *P* = 0.000).

## 4. Discussion

In the current prospective study, we further explored the prognosis significance of the NLR in patients with sepsis and found that the NLR measured at the time of admission to ICU was associated with 28-day mortality and correlated well with disease severity, according to APACHE II score. NLR was able to accurately stratify patients in terms of short-term mortality. These findings remained robust after adjusting for several potential covariates, suggesting that increased NLR was independently associated with unfavorable outcome in patients with sepsis. In our opinion, the strength of the NLR is the possibility of implementing this parameter simply by using already available biomarkers (neutrophil count and lymphocyte count). Therefore, this ratio is easy to integrate in clinical practice and cost effective.

Although the available information is still far from sufficient to comprehend thoroughly the economic burden of sepsis on an international scale, current studies demonstrate that sepsis has been a serious public health problem [15, 16]. The patients with septic shock have high risk of death, complications, and resource utilization [17]. Undoubtedly, the pivotal measure of improving outcome is to identify the septic patients with poor prognosis accurately [4]. Although recently introduced infection markers such as several cytokines and markers like soluble urokinase plasminogen activator receptor, endothelin-1, and copeptin have raised concerns in risk stratification and prognosis prediction, the application of these infection markers is still limited by validation, costs, and accessibility. To the best of our knowledge, immunocompetent leukocyte plays an important role in the systemic inflammatory response to infection. Most of the prognostic scores use leukocytosis (above 12.0 × 10^9^/L) or leukopenia (below 4.0 × 10^9^/L) as a severity index, but few consider the leukocyte subpopulations [14, 18]. Significant differences exist between circulating neutrophil and lymphocyte counts and, consequently, their ratio—referred to as the NLR—has been increasingly used in the prediction of the severity or prognosis in different clinical settings, including systemic inflammation and sepsis [19–21], ischemic events [22], and cancer [23, 24].

The cause responsible for NLR elevations correlating with poor outcome in patients with sepsis remains unclear, although there are a variety of plausible explanations. One of the most convincing explanations is based primarily on the physiological link between neutrophilia and lymphopenia with systemic inflammation and stress. The evolution of these leukocyte subpopulations may differ based on their respective role in the inflammatory response. Initially, Zahorec [9] explored the use of NLR in septic ICU patients and suggested that NLR was proposed as an indicator of the patient's response to inflammatory insult. Increased numbers of neutrophil implied that nidus of infection was not eradicated, which further induced depression of lymphocyte. Another large-scale study further found the presence of persistent lymphopenia and neutrophilia in trauma patients and patients who met the criteria for the systemic inflammatory response syndrome [25]. Evidence is growing that neutrophil is the key cellular component of host defense in the innate immune system against infectious injury, while lymphocyte is considered as the major cellular line of the adaptive immune system. Lymphocyte plays a key role in the regulation of inflammatory response, and their loss due to continuous sepsis-induced apoptosis may lead to the immune system suppression and nonresolution of inflammation [25, 26]. Taken together, the sustainability of infection and the incomplete eradication of nidus of infection are responsible for the increase of neutrophils production by the medulla and decrease lymphocytes counts by apoptosis and others mechanisms. Therefore, the resulting increase in NLR may identify patients who are in a state of nonresolution of inflammation, along with concomitant decreased survival rates.

We assessed the association between NLR and outcome in patients with sepsis. Similar to the findings of a previous clinical trial [9], our study clearly showed that the risk of death was associated with neutrophil count increase, lymphocyte count decrease, and subsequent increase in the NLR in the patients with sepsis at the time of admission to the ICU. In contrast, Salciccioli et al. found that there was no statistically significant relationship between NLR and mortality in patients with sepsis [13]. Another recent research indicated that the NLR on admission was significantly lower in patients who died before day 5 of septic shock onset than in survivors, and an increased NLR from day 1 to day 5 was associated with late death [12]. Why the results of these studies were conflicting can be attributed to the following reasons. First, the definition of primary outcome in different studies is inconsistent. Some of them defined 28-day mortality as outcome; nevertheless, some others defined early (before day 5 of septic shock onset) or late (on or after day 5 of septic shock onset) ICU mortality as outcome. Second, the disease severity of enrolled patients differs in the different studies. Some researches included both patients with sepsis and those with severe sepsis or septic shock when the other researches just enrolled patients with septic shock.

This study has a number of limitations that should be taken into consideration. First, we undertook a single-center observational study, and, as with any observational study, the potential remains for residual confounding. Thus, the results should be validated in other settings. Second, for some patients, repeated measurement data were available on the first day. We always used the first one and thus missed some information related to intraday cell count variations. Third, we just analyzed circulating neutrophil and lymphocyte counts and did not explore the different subpopulations of lymphocytes. The further work in our laboratory is to compare the subpopulations of lymphocytes, but the research data have not been shown in this study at present. Fourth, recently developed infection markers like soluble urokinase plasminogen activator receptor, endothelin-1, and copeptin were not evaluated and compared with NLR in the study. Finally, our sample size was so limited that the results should be further confirmed in a larger scale.

## 5. Conclusions

The NLR was associated with 28-day mortality in patients with sepsis. Use of the NLR may better help the physician stratify patients into prognostic categories. The present study can be considered as a preliminary attempt to clarify the strengths of NLR in prognosis prediction. However, the mechanisms underlying the association are yet to be fully elucidated and should be the focus of future prospective clinical research.

## Figures and Tables

**Table 1:** Baseline clinical and laboratory characteristics of the study subjects.

Characteristics	Patient group			
	All patients	Survivors	Nonsurvivors	*P* value
Demographics and underlying conditions				
Number of patients	333	253	80	—
Males, number (%)	188 (56.46%)	137 (54.15%)	51 (63.75%)	0.131
Age (years)	70.26 ± 15.79	67.82 ± 16.59	77.98 ± 9.49	0.000^*∗∗*^
COPD, number (%)	38 (11.41%)	23 (9.09%)	15 (18.75%)	0.017^*∗*^
Hypertension, number (%)	157 (47.15%)	115 (45.45%)	42 (52.50%)	0.271
CHD, number (%)	84 (25.23%)	55 (21.74%)	29 (36.25%)	0.008^*∗∗*^
Diabetes mellitus, number (%)	105 (31.53%)	80 (31.62%)	25 (31.25%)	0.950
Disease severity, number (%)				0.008^*∗∗*^
Sepsis	137 (41.14%)	117 (46.25%)	20 (25.00%)	—
Severe sepsis	149 (44.74%)	107 (42.29%)	42 (52.50%)	—
Septic shock	47 (14.11%)	29 (11.46%)	18 (22.50%)	—
Baseline parameters				
APACHE II score	11 (6–19)	9 (6–14.5)	20 (14–29)	0.000^*∗∗*^
WBC count (10^9^/L)	16.07 ± 6.63	15.35 ± 6.11	18.40 ± 7.67	0.000^*∗∗*^
Neutrophil (10^9^/L)	13.00 (9.80–17.55)	12.30 (9.53–16.76)	16.35 (11.16–20.14)	0.001^*∗∗*^
Lymphocyte (10^9^/L)	0.77 (0.51–1.30)	0.84 (0.54–1.40)	0.61 (0.35–0.87)	0.002^*∗∗*^
NLR	17.85 (9.61–28.19)	15.03 (8.94–24.67)	25.49 (16.64–47.15)	0.000^*∗∗*^
Platelet (10^9^/L)	191.24 ± 68.57	188.53 ± 61.15	199.96 ± 79.88	0.475
RBC count (10^9^/L)	4.10 ± 0.83	4.21 ± 0.79	3.74 ± 0.84	0.534
Hematocrit (%)	36.58 ± 6.59	37.34 ± 6.12	34.13 ± 7.43	0.066
RDW (%)	13.69 ± 1.87	13.40 ± 1.36	14.60 ± 2.79	0.000^*∗∗*^
Hemoglobin (g/L)	123.11 ± 22.99	126.35 ± 21.69	112.75 ± 24.13	0.290
PCT (ng/mL)	18.54 ± 7.28	17.89 ± 7.47	20.60 ± 7.13	0.521
CRP (mg/L)	100.05 ± 46.10	97.45 ± 47.43	108.26 ± 45.70	0.140
BNP (ng/mL)	173.0 (87.5–401.5)	160.0 (79.5–327.0)	181.0 (92.0–505.0)	0.020^*∗*^
Myoglobin (ng/mL)	83.8 (31.6–309.6)	71.4 (29.3–256.6)	153.0 (48.1–671.1)	0.011^*∗*^
CK-MB (ng/mL)	2.7 (1.4–6.0)	2.5 (1.2–5.0)	3.2 (2.1–9.0)	0.348
Troponin T (ng/mL)	0.03 (0.01–0.10)	0.02 (0.01–0.07)	0.08 (0.02–0.34)	0.009^*∗∗*^
BUN (mmol/L)	7.65 (5.13–11.70)	6.80 (4.64–10.30)	10.93 (7.70–19.20)	0.076
Scr (*μ*mol/L)	121.54 ± 50.63	109.45 ± 46.85	160.26 ± 67.97	0.004^*∗∗*^
Cystatin C (mg/L)	0.92 (0.00–1.51)	0.79 (0.00–1.32)	1.69 (1.02–2.74)	0.002^*∗∗*^
ALT (U/L)	29.0 (19.0–49.8)	28.0 (18.0–56.0)	30.0 (19.0–48.5)	0.075
AST (U/L)	36.0 (25.0–67.0)	35.0 (25.0–61.0)	46.0 (23.0–76.0)	0.056
Bilirubin (mg/dL)	22.61 ± 11.43	22.11 ± 10.92	24.19 ± 13.07	0.453
Albumin (g/L)	33.25 ± 6.04	34.25 ± 5.60	30.04 ± 6.32	0.000^*∗∗*^
FBG (mmol/L)	8.37 ± 5.08	8.40 ± 4.86	8.26 ± 5.12	0.830
Lactic acid (mmol/L)	2.0 (1.2–2.8)	1.8 (1.0–2.4)	2.3 (1.6–3.4)	0.002^*∗∗*^
Inflammatory cytokine				
IL-1B (pg/mL)	5.00 (5.00–5.12)	5.00 (5.00–5.00)	5.00 (5.00–6.04)	0.533
IL-2 receptor (U/mL)	1222 (801–1921)	1142 (764–1711)	1574 (1070–3379)	0.000^*∗∗*^
IL-6 (pg/mL)	27.9 (14.0–68.4)	26.1 (12.3–58.3)	49.8 (20.0–133.0)	0.052
IL-8 (pg/mL)	53.9 (21.3–153.5)	44.7 (18.7–139.0)	106.5 (35.1–242.8)	0.289
IL-10 (pg/mL)	5.9 (5.0–12.6)	5.7 (5.0–8.9)	9.0 (5.0–26.7)	0.099
TNF-*α* (pg/mL)	22.6 (15.9–34.4)	22.2 (15.6–33.7)	26.8 (16.6–41.2)	0.066
CD64	3.6 (1.6–6.2)	2.9 (1.5–5.8)	3.9 (1.7–6.3)	0.034^*∗*^
Site of infection, number (%)				0.602
Lung	184 (55.26%)	135 (53.36%)	49 (61.25%)	—
Abdomen	70 (21.02%)	56 (22.13%)	14 (17.50%)	—
Urinary tract	55 (16.52%)	44 (17.39%)	11 (13.75%)	—
Other	24 (7.21%)	18 (7.11%)	6 (7.50%)	—
Intervention, number (%)				
Mechanical ventilation	42 (12.61%)	17 (6.72%)	25 (31.25%)	0.000^*∗∗*^
Renal-replacement therapy	24 (7.21%)	10 (3.95%)	14 (17.50%)	0.000^*∗∗*^
Length of stay				
In the ICU (days)	4.5 (2–9)	4 (1–8)	6 (2–12)	0.041^*∗*^
In the hospital (days)	10 (7–14)	9 (7–13)	11 (7–16)	0.468

COPD: chronic obstructive pulmonary disorder; CHD: coronary heart disease; APACHE II: Acute Physiology and Chronic Health Evaluation II; WBC: white blood cell; NLR: neutrophil-to-lymphocyte ratio; RBC: red blood cell; RDW: red blood cell distribution width; PCT: procalcitonin; CRP: C-reactive protein; BNP: brain natriuretic peptide; CK-MB: creatine kinase-MB; BUN: blood urea nitrogen; Scr: serum creatinine; ALT: alanine transaminase; AST: aspartate transaminase; FBG: fasting blood glucose; IL: interleukin; TNF-*α*: tumor necrosis factor-*α*.

Data are expressed as number (%), mean (standard deviation, SD), or median (interquartile range, IQR) as appropriate.

Significant differences are marked by ^*∗*^(*P* < 0.05) or ^*∗∗*^(*P* < 0.01).

**Table 2:** Performance of variables in predicting unfavorable outcome.

Variables	AUC ROC	*P* value	Cutoff value	Sensitivity (%)	Specificity (%)
APACHE II score	0.828 ± 0.026	0.000^*∗∗*^	≥16.5	76.3	70.8
Neutrophil	0.633 ± 0.036	0.000^*∗∗*^	≥14.2	73.8	45.8
Lymphocyte	0.650 ± 0.035	0.000^*∗∗*^	≤0.64	75.0	58.1
NLR	0.695 ± 0.036	0.000^*∗∗*^	≥23.8	81.3	53.6

AUC ROC: area under the receiver operating characteristic curve; APACHE II: Acute Physiology and Chronic Health Evaluation II; and NLR: neutrophil-to-lymphocyte ratio.

Significant differences are marked by ^*∗∗*^(*P* < 0.01).

**Table 3:** Univariate odds ratios of variables for predicting unfavorable outcome.

Variables	Standard *β* value	OR	95% CI	*P* value
APACHE II score	0.2342	1.168	1.102–1.238	0.000^*∗∗*^
Neutrophil	−0.0875	0.916	0.766–1.096	0.339
Lymphocyte	0.0671	1.069	0.808–1.416	0.639
NLR	0.0378	1.038	1.008–1.070	0.013^*∗*^
WBC	0.0195	1.020	0.955–1.089	0.558
Lactic acid	0.0461	1.047	0.860–1.276	0.647
Age	0.0813	1.085	1.039–1.132	0.000^*∗∗*^

APACHE II: Acute Physiology and Chronic Health Evaluation II; NLR: neutrophil-to-lymphocyte ratio; and WBC: white blood cell.

The OR indicates the risk of obtaining unfavorable outcome. Standard *β* value was calculated using the semistandardization method (*X* standardization).

Significant differences are marked by ^*∗*^(*P* < 0.05) or ^*∗∗*^(*P* < 0.01).

**Table 4:** Independent predictors of unfavorable outcome by multivariate logistic regression analysis.

Variables	Standard *β* value	OR	95% CI	*P* value
APACHE II score	0.2639	1.168	1.108–1.230	0.000^*∗∗*^
NLR	0.0471	1.043	1.012–1.083	0.016^*∗*^
Age	0.0745	1.077	1.034–1.122	0.000^*∗∗*^

APACHE II: Acute Physiology and Chronic Health Evaluation II; NLR: neutrophil-to-lymphocyte ratio.

The OR indicates the risk of obtaining unfavorable outcome. Standard *β* value was calculated using the semistandardization method (*X* standardization). Variables not listed in the table were removed from the stepwise analysis.

Significant differences are marked by ^*∗*^(*P* < 0.05) or ^*∗∗*^(*P* < 0.01).

**Table 5:** Correlations of indicators with disease severity.

Variables	Sepsis	Severe sepsis	Septic shock	*P* value
APACHE II score	8.00 (5.00–11.50)	13.00 (9.00–19.00)	21.50 (14.75–30.25)	0.000^*∗∗*^
Neutrophil	11.75 (9.52–16.65)	13.35 (10.44–18.22)	15.98 (10.14–21.29)	0.016^*∗*^
Lymphocyte	1.12 (0.71–1.70)	0.67 (0.45–0.95)	0.51 (0.26–0.78)	0.000^*∗∗*^
NLR	11.11 (6.98–18.24)	22.67 (12.35–31.89)	31.50 (22.56–46.94)	0.000^*∗∗*^

APACHE II: Acute Physiology and Chronic Health Evaluation II; NLR: neutrophil-to-lymphocyte ratio.

Significant differences are marked by ^*∗*^(*P* < 0.05) or ^*∗∗*^(*P* < 0.01).

## Abbreviations

NLR: Neutrophil-to-lymphocyte ratio
ICU: Intensive care unit
APACHE II: Acute Physiology and Chronic Health Evaluation II
SD: Standard deviation
IQR: Interquartile range
ROC: Receiver operating characteristic
AUC: Area under the curve
WBC: White blood cell
PCT: Procalcitonin
CRP: C-reactive protein
OR: Odds ratio
CI: Confidence interval.
